# The Induction Effect of Am80 and TSA on ESC Differentiation via Regulation of *Stra8* in Chicken

**DOI:** 10.1371/journal.pone.0140262

**Published:** 2015-11-25

**Authors:** Yani Zhang, Qisheng Zuo, Zhiyong Liu, Dong Li, Beibei Tang, Tian-rong Xiao, Chao Lian, Yingjie Wang, Kai Jin, Yilin Wang, Wenhui Zhang, Bichun Li

**Affiliations:** 1 College of Animal Science and Technology, Yangzhou University, Yangzhou 225009, China; 2 Key Laboratory of Animal Breeding Reproduction and Molecular Design for Jiangsu Province, Yangzhou 225009, China; Qingdao Agricultural University, CHINA

## Abstract

*Stra8* encodes stimulated by retinoic acid gene 8, a protein that is important for initiation of meiosis in mammals and birds. This study was aimed at identifying the active control area of chicken *STRA8* gene core promoter, to screen optimum inducers of the *STRA8* gene, thus to enhance the differentiation of embryonic stem cells (ESCs) into spermatogonial stem cells. Fragments of chicken *STRA8* gene promoter were cloned into fluorescent reporter plasmids and transfected into DF-1 cells. Then Dual-Luciferase® Reporter Assay System was used to identify the activity of the *STRA8* gene under different inducers. Our studies showed that the promoter fragment −1055 bp to +54 bp of Suqin chicken Stra8 revealed the strongest activity. The dual-luciferase® reporter showed that Tamibarotene (Am80) and TrichostatinA (TSA) could significantly enhance STRA8 transcription. The in vitro inductive culture of chicken ESCs demonstrated that spermatogonial stem cells (SSC)-like cells appeared and Integrinβ1 protein was expressed on day 10, indicating that Am80 and TSA can promote ESCs differentiation into SSCs via regulation of Stra8.

## Introduction

Stimulated by retinoic acid gene 8 (*Stra8*) is a specific gene that is expressed in mammalian germ cells during transition from mitosis to meiosis and plays a key role in the initiation of meiosis in mammals and birds. When *Stra8* is deleted, germ cells are unable to complete meiosis [1, 2]. Germ cells in male rats in which *Stra8* is deleted cannot enter meiotic prophase. Female germ cells also do not show features of meiosis in the absence of *Stra8*, indicating that *Stra8* plays a key role in the initiation of meiosis [3]. Only germ cells are activated to express *Stra8* by retinoic acid (RA) and then enter the meiosis phase from mitosis [4–7]. The research have show that RA and Stra8 gene combination can promote the male ESC differentiation to germ cell direction synactic that was proved by both significant cellular morphological changes and also the highly expressed germ cell marker genes[8].

The biological activity of RA is mediated through the retinoic receptor (RAR) and retinoid receptor (RXR) [9]. RARs and RXRs are encoded by different genes, which each produce α, β, and γ subtypes. The physiological function of RA is regulated by different RAR isoforms, which combine with RXRs to form dimers. RXR may play a role as a scaffold protein to promote the binding of DNA to the receptor. However, a variety of coactivators and corepressors may bind to RARE and hinder the formation of the RAR-RXR-RA compound [10]. In the absence of RA, RAR/RXR dimers recruit corepressor complexes containing histone deacetylase (HDAC) activity and induce transcriptional repression [11]. In the presence of RA, RA can bind with RAR/RXR dimers, followed by alteration of the corepressor by an HDAC activity coactivator, which can induce the transcriptional activation and epigenetic modification that occurs with RA-responsive gene induction and inhibition [12].

Tamibarotene (Am80) is an activator of RARα that can specifically bind with RARα [13]. The activity of Am80 is about ten times that of all trans-retinoic acid (ATRA). HDAC can bind with RARE of the *Stra8* promoter, causing chromatin condensation and subsequent silencing of gene transcription [14]. Trichostatin A (TSA) can bind with HDAC, which inactivates HDAC. ATRA is the common inducer in research involving *Stra8*, although we do not know whether the other inducers can induce the expression of *Stra8*. Thus, the purpose of this study was to use bioinformatics analysis and the dual luciferase activity detection system to search for regulatory elements that are associated with the transcription of *Stra8*, to test the influence of Am80 and TSA on the promoter activity of *Stra8* and embryonic stem cell (ESC) differentiation, and to provide a reference for screening for the inducer of *Stra8*.

## Results

### Comparison of the activity of fragments of different lengths of the chicken *Stra8* promoter and analysis of transcriptional regulatory elements

**Fig 1:**
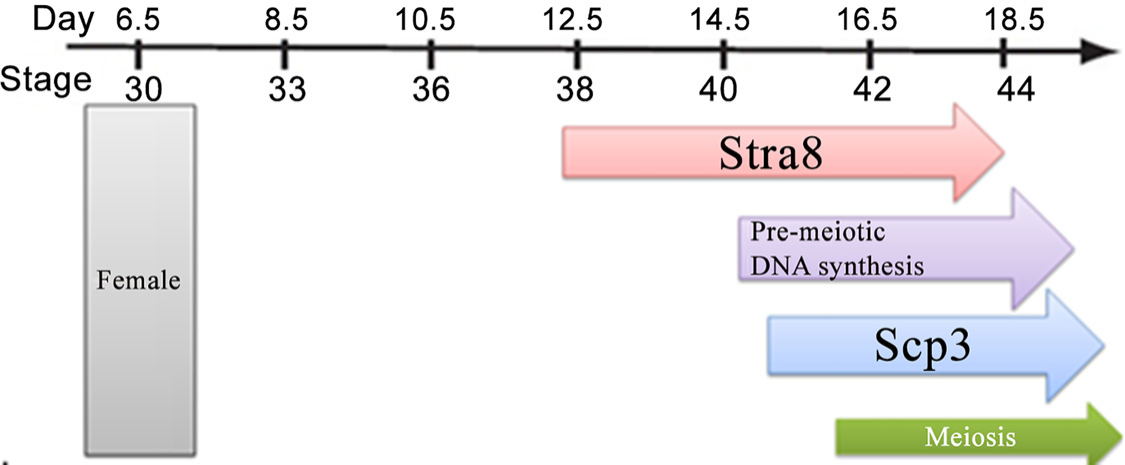
Schedule of meiosis-related marker genes during chicken ovarian development.

**Fig 2:**
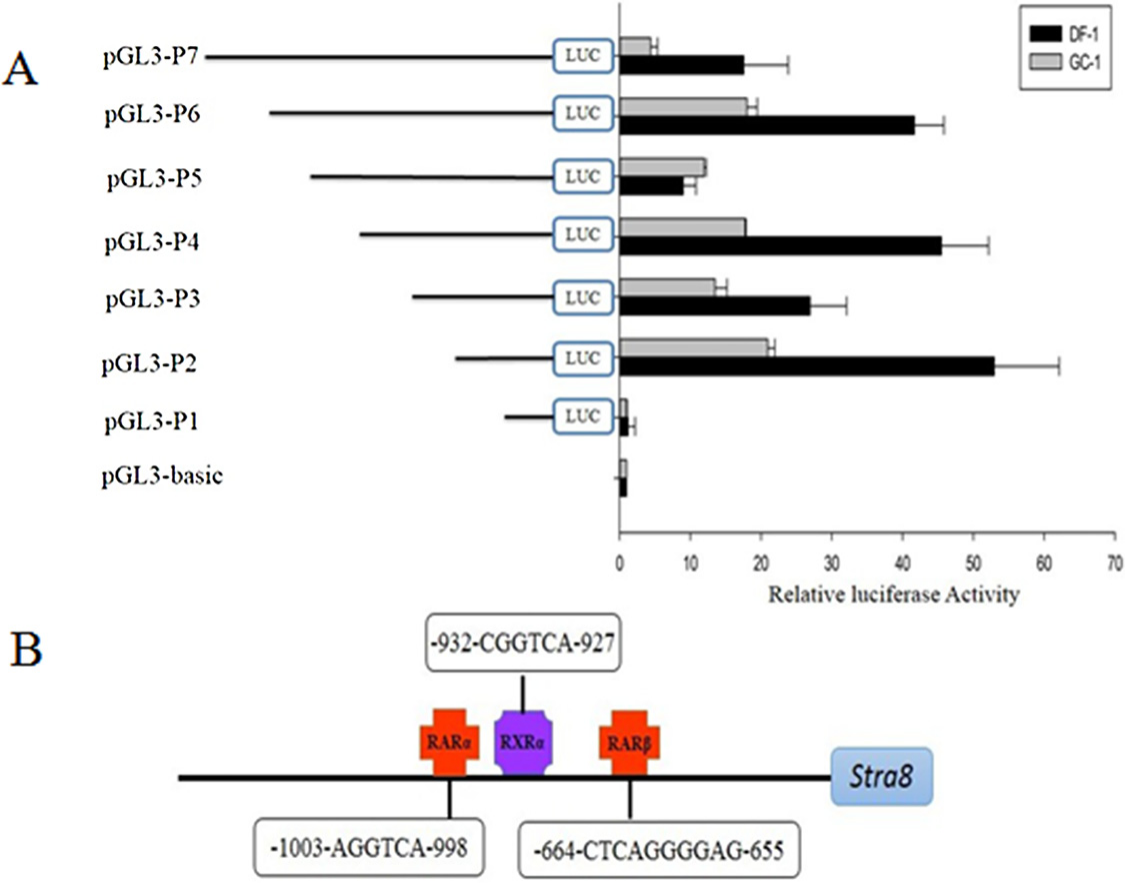
Activity of the deletion fragments of the chicken *Stra8* promoter and binding sites for regulatory elements at −1955 to +45 bp. A: Activity of the deletion fragment of the chicken *Stra8* promoter in DF-1 and GC-1 cells. B: Prediction of binding sites for regulatory elements at −1955 to +45 bp of *Stra8*.C:The result of *Stra8*- EGFP transfection in DF-1.Left:The blank group. Mid: Transfected with N1-EGFP. Right,Transfected with *Stra8*-EGFP.

Studies have shown in our laboratory that Stra8 plays a key role in the initiation of meiosis in birds chicken (Fig 1). To analyze the promoter activity of the core region of *Stra8*, the DF-1 and GC-1 cell lines were transfected with recombinant plasmids and the pRL-SV40 plasmid (as an internal control). The pGL3-basic plasmid was used as a negative control. The promoter activity was detected with the dual-luciferase assay. The promoter activity of chicken *Stra8* showed the same trend in these two cell lines. The recombinant plasmid pGL3-P2 (−500/+54) had the highest promoter activity. Almost no difference was seen between pGL3-P4 (−1055/+54) and pGL3-P2 (−500/+54). The promoter activity of pGL3-P1 (−201/+54) was sharply decreased compared to pGL3-P2 (−500/+54), indicating that the core promoter region was between −554 bp and −255 bp (Fig 2A).

The regulatory elements RARα, RXRα, and RARβ were identified by TFsearch and AliBaba online software (Fig 2B). Combined with the activity of different promoter fragments, the recombinant plasmid pGL3-P4 (−1055/+54) was selected to construct the recombinant plasmid *Stra8*-EGFP, and we find that *Stra8*-EGFP can express green fluorescent protein, it shows that the fragment in −1055/+54 of *Stra8* promoter can promote the expression of genes(Fig 2C).

### Screening of inducers based on the transcriptional regulatory elements of the chicken *Stra8* promoter

**Fig 3:**
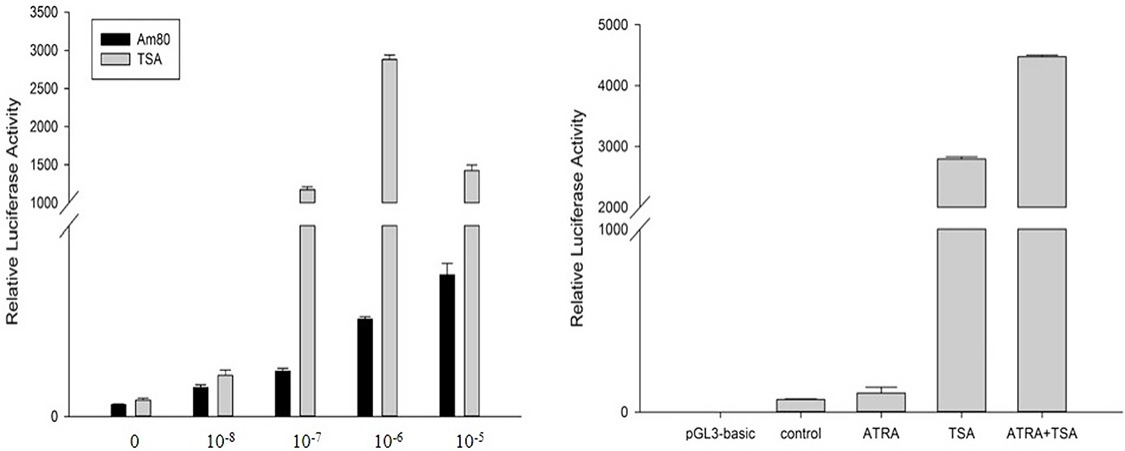
Effects of the induction of Am80 and TSA on the *Stra8* promoter activity. A: Effect of different concentrations of Am80 and TSA on *Stra8* promoter activity. B: Effect of Am80 (10^−5^ mol/L) or TSA (10^−6^ mol/L) on *Stra8* promoter activity. **P < 0.01.

Am80 is an activator of RARα and can specifically combine with RARα. TSA can combine with HDAC and inactivate HDAC. The recombinant plasmid pGL3-P4 (−1055/+54) was transfected into DF-1 cells, which were treated with different concentrations of Am80 and TSA. The reporter activity was the highest when the concentrations of Am80 and TSA were 10^−5^ mol/L and 10^−6^ mol/L, respectively (Fig 3A). Because the functions of Am80 and TSA are different, DF-1 cells were transfected with pGL3-P4 (−1055/+54) and treated with Am80 and TSA alone or together. The reporter activity was increased significantly when Am80 and TSA were added together; treatment with both compounds induced a 94-fold increase compared with ATRA induction (Fig 3B). All the result showed that Am80 and TSA can increase the promoter activity of *Stra8*.

### The regulatory function of Am80 and TSA for *Stra8*

**Fig 4:**
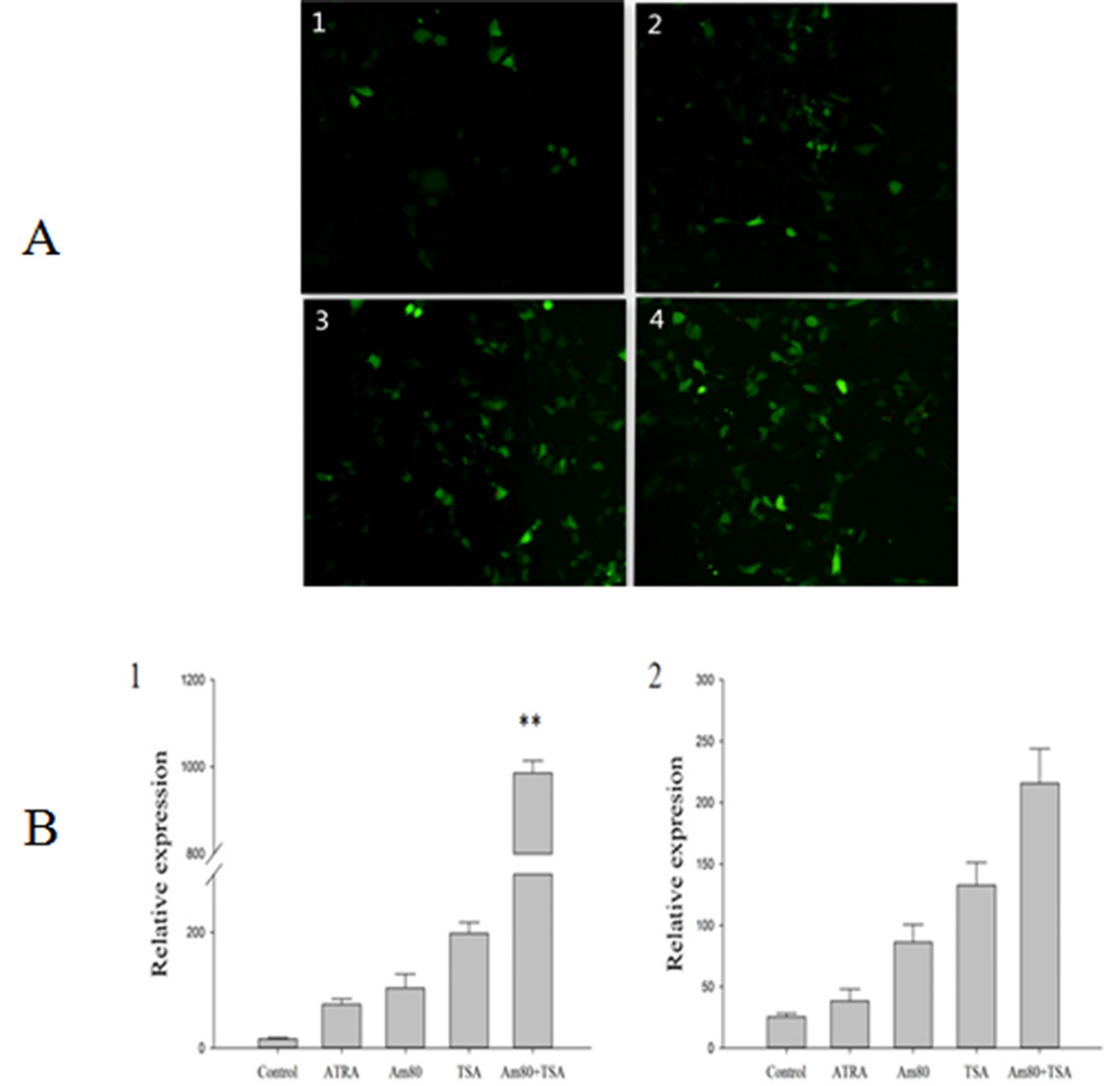
The regulatory function of Am80 and TSA induction on the expression of *Stra8*. A: 1. ATRA induction group; 2. Am80 induction group; 3. TSA induction group; 4. Am80 and TSA combination induction group. B: 1: *Stra8*; 2: Scp3. **P < 0.01.

DF-1 cells were treated with ATRA, Am80, or TSA after the cells were transfected with *Stra8*-EGFP for 4 hours. After 36 hours of induction, the cells were observed under a fluorescence microscope. The highest green fluorescence intensity was obtained when Am80 and TSA were used together for induction (Fig 4A). The trend in fluorescence intensity for the different treatment groups was consistent with the relative luciferase values, suggesting that Am80 and TSA can induce the expression of *Stra8* at the cellular level as well.

The left ovary was removed when the embryo was hatched into 12.5 days, and the ovary was cultured in tissue culture medium for 48 hours. During the culture process, an inducer was added to the different experimental groups. RNA was extracted from the ovary, and the meiotic marker genes *Stra8* and Synaptonemal complex protein 3(Scp3) were detected with quantitative real-time reverse transcriptase PCR (qRT-PCR). The expression levels of *Stra8* and Scp3 following treatment with Am80 or TSA were significantly higher than with ATRA, regardless of whether Am80 and TSA were used individually or together (Fig 4B), indicating that Am80 and TSA can be used as inducers to enhance the promoter activity of *Stra8*.

### ESC differentiate following Am80 and TSA induction

**Fig 5:**
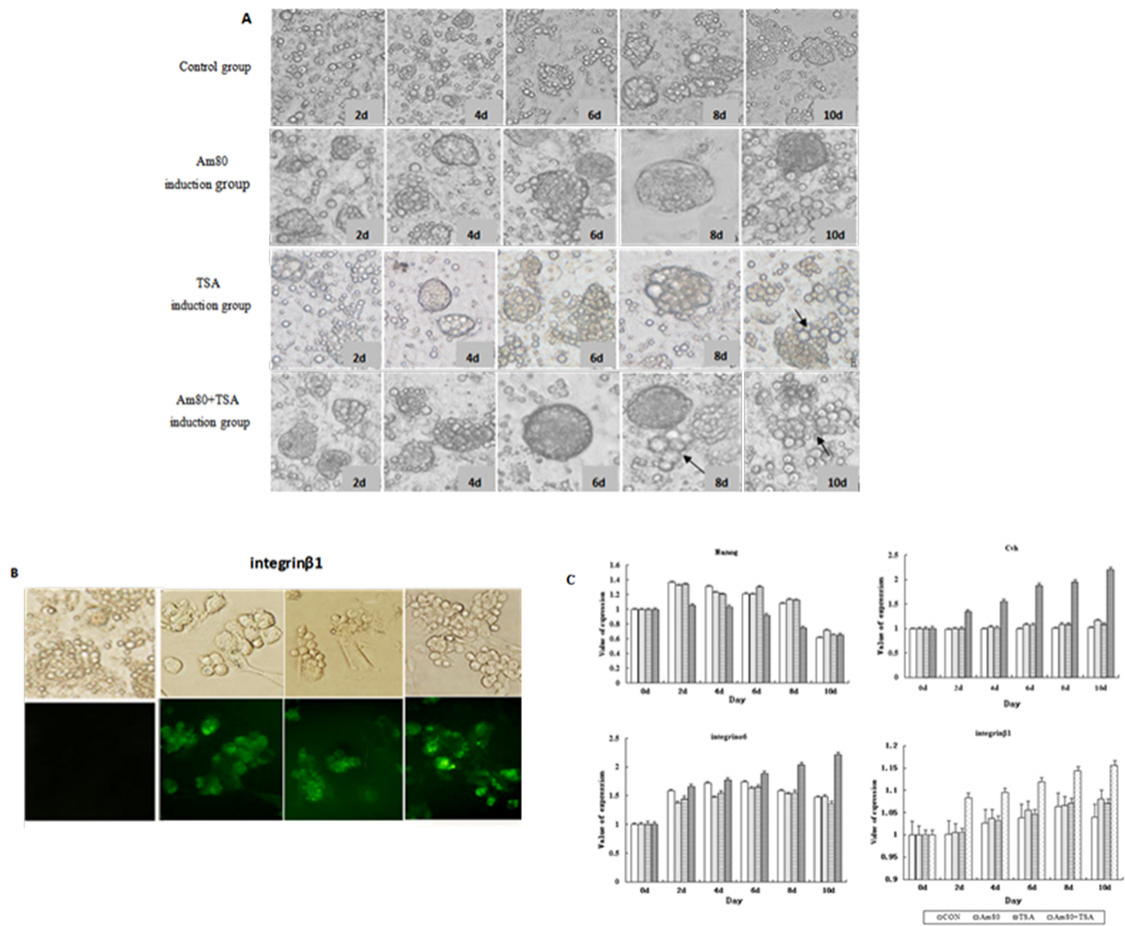
The effect of Am80 and TSA on ESC differentiation. A: Differentiation of chicken ESCs into SSCs is induced by Am80 and TSA; B: Immunohistochemical detection of integrinβ1 protein expression in chicken ESC treated with Am80 and TSA; C: qRT-PCR detection of specific marker genes for germ cells at different days after Am80 and TSA induction.

The in vitro induction experiment showed that morphological changes were induced in chicken ESC with ATRA(10^−5^ mol/L), Am80 (10^−5^ mol/L), and TSA (10^-6^mol/L). ESC grew slowly in basic medium. Two or three cells grouped together on the 2^nd^ day of culture, a few cells began to conglomerate on the 4^th^ day of culture, a small colony appeared on the 6th day of culture, and the number of cell colonies was increased by the 8th day culture. For Am80 and TSA induction, a few embryoid bodies appeared on the 4^th^ day of induction and became large in volume by the 8^th^ day of induction. A few SSC-like cells were observed by the 10^th^ day. However, in the Am80 + TSA induction group, a few SSC-like cells appeared on the 8^th^ day of induction, and the number of SSC-like cells were increased on the 10th day (Fig 5A). Integrinβ1 protein was expressed on the 10^th^ day of induction (Fig 5B), indicating that Am80 and TSA can promote the differentiation of ESC into SSCs. The specific marker gene of germ cells was detected during the different induction days, demonstrating that Am80 and TSA can promote ESC differentiation into SSC-like cells. More SSC-like cells were observed with a combination of induction by Am80 and TSA compared to induction with an individual compound (Fig 5C).qRT—PCR results showed that expression of integrinβ1 and integrinα6 in the Am80 group, TSA group and Am80+TSA group were significantly increased compared with control group, and Am80+TSA group was aslo significantly increased than Am80 group and TSA group,It suggests that treatment of Am80 and TSA can promote the ESC differentiation into SSC-like.

## Discussion

*Stra8* is an important gene for induction of differentiation of mouse P19 teratocarcinoma cells by ATRA [15], and *Stra8* plays an key role in the process of induction of meiosis and normal meiotic prophase. ATRA is the natural isomer of RA and is mainly metabolized to active products through isomerases such as 11cis-RA, 13cis-RA, and 9cis-RA. Cis-retinoic can bind with RARs and regulate cell proliferation and differentiation at the mRNA level [16]. Stra8 gene performed function through the six ligand with RA, including three RAR transcription factor (RARα, RARβand RARγ) and three RXR transcription factor (RXRA and RXRB and RXRG) [17,18].Stra8 can be activated to enter into the nucleus by bindding RAR receptor, and then start meiosis of sperm through specific gene[19]. Mice which knocked out RARγ and RARα receptor have a low expression of Stra8 gene and appear VAD-like phenotype[20–22].Tsutomu Endo et al. firstly found that the lack of Stra8 gene and RA can stimulte undifferentiated spermatagonial stem cells in mice to Sperm Cells [23]

Am80 and TSA were used to explore regulation of the *Stra8* promoter in this study. Am80 is an active metabolic product of vitamin A and an analog of ATRA, which can specific to combine with RARα receptor[24–26], it aslo can induce RARα to express[27], it has reported that approximately 10-fold greater effective biological activity than ATRA[28]. Histones are required for chromosomal folding, and the degree of combination (loose or close) is thought to be a key factor in transcription factor binding to promoters [29]. In the nucleus, histone acetylation and histone deacetylation occur in a dynamic balance and are regulated by histone acetyltransferase (HAT) and HDAC [30]. TSA is the most effective inhibitor of HDAC [31]. It has been reported that treatment with the inhibitor of histone deacetylases activity (trichostatin A; TSA) in cloned embryos of mouse, rabbit, and pig can regulate acetylation levels of certain histone residues[32],it aslo can improve embryo to develop to the blastocyst stage [33–38],and increase the number of live offspring after transfer to foster mothers[39–41].

TSA binds to HDAC and inhibits its activity, allowing the RAR/RXR dimer to recruit HAT. This leads to auxiliary activation and results in epigenetic modification that induces gene expression via chromatin remodeling. In this study, DF-1 cells were transfected with the recombinant plasmid pGL3-P4 and treated with Am80 and ATRA. The relative luciferase activity values of the Am80-treated group were higher than with ATRA induction. When Am80 and TSA were used together, the relative luciferase activity values were much higher than with ATRA induction. Meanwhile, DF-1 cells were transfected with the recombinant plasmid pEGFP-*Stra8* and treated with ATRA and TSA alone or in combination, the green fluorescent protein (GFP) expression intensity in the group treated with both Am80 and TSA was higher than in the cells treated with ATRA alone. These results show that the synergistic effect of Am80 and TSA can significantly regulate the activity of the *Stra8* promoter.

When ESC were treated with Am80 and TSA, germ cells were readily generated. Integrinβ1 protein was expressed on the 10^th^ day of induction, and the specific marker gene of germ cells was detected after different days of induction, demonstrating that Am80 and TSA can promote ESC to differentiate into PGC-like and SSC-like cells and that both compounds were effective inducers, which may improve the efficiency of generation of germ cells.

## Conclusion

In this study, the regulatory elements (RAR and RXR) of the *Stra8* promoter were identified with the dual luciferase reporter gene assay system and bioinformatics analysis. The regulatory function of Am80 and TSA at the *Stra8* promoter was verified. The optimal induction concentrations of Am80 and TSA were 10^−5^ mol/L and 10^−6^ mol/L, respectively. Am80 and TSA can promote ESC to differentiate into PGC-like and SSC-like cells.

## Materials and Methods

### Ethics Statement

Procedures involving animals and their care conformed to the U.S. National Institute of Health guidelines (NIH Pub. No. 85–23, revised 1996) and were approved by the laboratory-animal management and experimental-animal ethics committee of Yangzhou University. The Suqin yellow chickens used in this study were provided by the Institute of Poultry Science, Chinese Academy of Agriculture Sciences. The animal procedures were approved by the Institutional Animal Care and Use Committee of Yangzhou University.

### Materials

The pEGFP-N1 vector was constructed in our laboratory. *E*. *coli* DH5α competent cells, gel extraction kit, and miniprep kits were purchased from the Tiangen Company (Beijing, China). DL5000 DNA Marker Prime STARMax DNA Polymerase, T4 DNA ligase, and restriction endonuclease were purchased from Takara (Dalian, China). The expression vector pGL3.0-Basic, pRL-SV40, and the Dual-Luciferase Reporter Assay System were purchased from Promega Corporation. LipofectamineTM2000 was purchased from Invitrogen Corporation. ATRA, Tamibarotene (Tamibarotene, Am80), and TSA were purchased from SIGMA Company. The chicken embryo fibroblast cell line, DF-1, and Mouse spermatogonial cell line, GC-1,were purchased from ATCC. Primer synthesis and sequencing were conducted by the Invitrogen Company of Shanghai (China).

### Isolation and cultivation of chicken ESC

The blastodermal cells at stage X Suqin yellow chicken embryos were collected using the spoon method, transferred to tissue culture dishes, and rinsed with Ca^2+^- and Mg^2+^-free phosphate-buffered saline (PBS) to remove the yolks and vitelline membrane. After washing with PBS, ESC were transferred into a fresh centrifuge tube and then gently mechanically dissociated. The dissociated cells were collected by centrifugation at 1000 rpm for 8–10 min and suspended in ESC complete medium. The cells were seeded on inactivated chicken embryonic fibroblasts (CEF) feeder cells and incubated at 37.0°C in 5% CO_2_. The medium was replaced daily with fresh medium. After 5–6 days, the cell colonies with undifferentiated morphology were rinsed three times with PBS and digested at 37.0°C for 2–3 min with 0.05% Trypsin-EDTA (Gibco, UK). The dissociated ESC clusters were suspended with pipetting and sub-cultured at 37.0°C in 5% CO_2_ on the feeder cell layer. Half the medium was replaced daily with fresh medium.

#### Analysis of bioinformatics

The online TFsearch software programs (http://www.cbrc.jp/research/db/TFSEARCH.html) and (http://www.gene-regulation.com/pub/programs/alibaba2/index.html) were used to predict and analyze the promoter region and potential transcription factor binding sites of *Stra8*.

### Construction of expression vectors for a series of *Stra8* promoter deletions and *Stra8*-EGFP

A series of *Stra8* promoter deletions which were cloned from the genome of chicken and pGL3-basic were digested with *Kpn*I and *Hin*dIII, respectively. The purified enzyme fragment was ligated with T4 DNA ligase and transformed into DH5α cells. The vectors were verified with double enzyme digestion and sequencing. The new plasmids were named pGL3-P1 (−201/+54), pGL3-P2 (−500/+54), pGL3-P3 (−739/+54), pGL3-P4 (−1055/+54), pGL3-P5 (−1209/+54), pGL3-P6 (−1629/+54), and pGL3-P7 (−1901/+54). pGL3-P4 (−1055/+54) was used as the template for PCR. Both of the PCR product and pEGFP-N1 were digested with *Ase*I and *Hin*dIII then connected by DNA ligase to construct *Stra8*-EGFP. primers used to construct the Stra8 vectors refer to S1 Table.

### Cell transfection and dual-luciferase assay

DF-1 or GC-1 cells (2.5 × 10^5^) were plated in 24-well plates before transfection. The cells were grown to 80–90% confluency and transfected with Lipofectamine 2000. The ratio of reconstruction plasmid and pRL-SV40 was 25:1, and pGL3-basic was used as the negative control. After transfection for 6 hours, Am80 and TSA were added at various concentrations. ATRA (10^−6^ mol/L) was used as the control. After induction for 48 hours, the Dual-Luciferase^®^Reporter Assay was performed. Cells were collected in PBS and placed in 96-well plates. After 15 min, 100 μL Dual-Glo® Luciferase Buffer was added to detect the firefly luciferase activity values, and then 100 μL Dual-Glo® Stop & Glo® Buffer was added to obtain the renilla luciferase activity values. The relative luciferase activity for the promoter activity was calculated as the firefly luciferase value/renilla luciferase value. All assays were repeated three independent times, and results are shown with standard errors.

### ESC differentiate following Am80 and TSA induction

To validate the induction effect of Am80 and TSA, chicken ESC were seeded into 24-well plates at a density of 2 × 10^5^ cells per well. Four groups of cells were studied: (1) cells grown in basic DMEM (Control group), (2) cells grown in normal DMEM and treated with ATRA (10^−5^ mol/L) induction, (3) cells treated with Am80 (10^−5^ mol/L) induction, (4) cells treated with TSA (10^−6^ mol/L) induction, and (5) cells treated with both Am80 (10^−5^ mol/L) + TSA (10^−6^ mol/L) induction. The cells were observed every 2 days by microscope after treatment. The induction experiments were done using three replicates.

### qRT-PCR

Total RNA was extracted with Trizol Reagent after 2, 4, 6, 8, and 10 days of induction. cDNA was synthesized by using the Revert Aid first-strand cDNA synthesis kit (ChinaGen Biological Technology). qRT-PCR was performed using SYBR green PCR Master Mix (ChinaGen Biological Technology) on an ABI 7300 instrument. The mRNA level in ESC was normalized to the level of β-actin. PCR was performed in duplicate, and error bars in charts represent the corresponding standard deviations.

### Immunofluorescence Assay

Cell suspensions were allowed to adhere to poly-l-lysine-coated slides and fixed for 10 min in 4% paraformaldehyde. After blocking in 10% bovine serum albumin (BSA) in PBS for 1 h, antibodies against anti-**integrin β1** (Millipore) were added at a 1:200 dilution in 10% BSA in PBS and incubated overnight at 4°C. After washing in 0.05% Tween-20 in PBS three times, FITC-labeled secondary antibodies (Biosynthesis Biotechnology CO., LTD.) were added at a 1:100 dilution for 2 h. After washing again with 10% BSA-PBS, cells were observed under an inverted fluorescence microscope (IX51 from Olympus).

### Statistical analysis

All data are presented as the mean ± SEM. Statistical significance was evaluated using a one-tailed unpaired Student’s t-test with GraphPad Prism5 statistics software (*: *P* ≤ 0.05, **P ≤ 0.01).

## Supporting Information

S1 Table The sequences of primers used to construct the *Stra8* vectors. (DOCX)
