# Supplementary material for: The Induction Effect of Am80 and TSA on ESC Differentiation via Regulation of Stra8 in Chicken
Source: PLoS One. 2015 Nov 25;10(11):e0140262. doi: 10.1371/journal.pone.0140262 (PMC4659672; doi:10.1371/journal.pone.0140262)
Supplement: S1 Table — (DOCX) [file pone.0140262.s001.docx]

**Table 1. The sequences of primers used to construct the *Stra8* vectors**

| Name | Sense primer (5'→3') | Annealing temp (°C) | Size (bp) |
| --- | --- | --- | --- |
| pGL3-P1  (−201/+54) | F: GG***GGTACC***GGGAGCAAAGCTGCGTCC  R: CC***AAGCTT***CCCGTTACCAATTGCACGTA | 61.5 | 255 |
| pGL3-P2  (−500/+54) | F: GG***GGTACC***GGTCCGCCTTGATCTCCG  R: CC***AAGCTT***CCCGTTACCAATTGCACGTA | 60.5 | 554 |
| pGL3-P3  (−739/+54) | F: GG***GGTACC***ATTAGCGAGCGGCACGAAG  R: CC***AAGCTT***CCCGTTACCAATTGCACGTA | 60.5 | 793 |
| pGL3-P4  (−1055/+54) | F: GG***GGTACC***TCGATACAGGCTGGTTTTCAG  R: CC***AAGCTT***CCCGTTACCAATTGCACGTA | 58.5 | 1109 |
| pGL3-P5  (−1209/+54) | F: GG***GGTACC***ATCACATAAGGACTGCCCGA  R: CC***AAGCTT***CCCGTTACCAATTGCACGTA | 60.5 | 1263 |
| pGL3-P6  (−1629/+54) | F: GG***GGTACC***CCAAATTAAGCTCCAGGCAA  R: CC***AAGCTT***CCCGTTACCAATTGCACGTA | 58.5 | 1683 |
| pGL3-P7  (−1901/+54) | F: GG***GGTACC***CTGGGAACGAAACAATCTCAG  R: CC***AAGCTT***CCCGTTACCAATTGCACGTA | 59 | 1955 |
| Stra8-EGFP | F:CCG**ATTAAT**TCGATACAGGCTGGTTTTCAG  R:CC**AA GCTT**CCCGTTACC AATTGCACGTA | 58 | 1109 |

The underlined letters indicate the restriction enzyme sites for *Kpn*I and *Hin*dIII to construct the

deletion fragment of *Stra8* promoter. And VspⅠand Hind Ⅲ for *Stra8-*EGFP
